# Comparison of whole-genome prediction models for traits with contrasting genetic architecture in a diversity panel of maize inbred lines

**DOI:** 10.1186/1471-2164-13-452

**Published:** 2012-09-04

**Authors:** Christian Riedelsheimer, Frank Technow, Albrecht E Melchinger

**Affiliations:** 1 Institute of Plant Breeding, Seed Science and Population Genetics, University of Hohenheim, Stuttgart, Germany

**Keywords:** Genomic selection, Whole-genome prediction, Genetic architecture, Complex traits, *Zea mays*

## Abstract

**Background:**

There is increasing empirical evidence that whole-genome prediction (WGP) is a powerful tool for predicting line and hybrid performance in maize. However, there is a lack of knowledge about the sensitivity of WGP models towards the genetic architecture of the trait. Whereas previous studies exclusively focused on highly polygenic traits, important agronomic traits such as disease resistances, nutrifunctional or climate adaptational traits have a genetic architecture which is either much less complex or unknown. For such cases, information about model robustness and guidelines for model selection are lacking. Here, we compared five WGP models with different assumptions about the distribution of the underlying genetic effects. As contrasting model traits, we chose three highly polygenic agronomic traits and three metabolites each with a major QTL explaining 22 to 30% of the genetic variance in a panel of 289 diverse maize inbred lines genotyped with 56,110 SNPs.

**Results:**

We found the five WGP models to be remarkable robust towards trait architecture with the largest differences in prediction accuracies ranging between 0.05 and 0.14 for the same trait, most likely as the result of the high level of linkage disequilibrium prevailing in elite maize germplasm. Whereas RR-BLUP performed best for the agronomic traits, it was inferior to LASSO or elastic net for the three metabolites. We found the approach of genome partitioning of genetic variance, first applied in human genetics, as useful in guiding the breeder which model to choose, if prior knowledge of the trait architecture is lacking.

**Conclusions:**

Our results suggest that in diverse germplasm of elite maize inbred lines with a high level of LD, WGP models differ only slightly in their accuracies, irrespective of the number and effects of QTL found in previous linkage or association mapping studies. However, small gains in prediction accuracies can be achieved if the WGP model is selected according to the genetic architecture of the trait. If the trait architecture is unknown *e.g.* for novel traits which only recently received attention in breeding, we suggest to inspect the distribution of the genetic variance explained by each chromosome for guiding model selection in WGP.

## Background

Whole-genome prediction (WGP) is expected to reshape plant breeding fundametally in the near future [1-3]. Whereas the approach has been initially proposed [4] and rapidly implemented in animal breeding [5], recent empirical studies demonstrated also its potential in hybrid maize breeding [6-8]. Recently, we showed that WGP allows a reliable screening of large germplasm collections of diverse maize inbred lines for their potential to create superior hybrids [9]. However, these studies exclusively focused on predicting highly polygenic traits such as grain yield or biomass accumulation with genetic architectures close to the infinitesimal genetic model [10].

In maize, several economically important traits are genetically less complex with few quantitative trait loci (QTL) explaining a large proportion of the genetic variance. Examples include pest and disease resistances or nutrifunctional compounds such as bioavailable minerals [11] or *β*-carotene [12]. In addition, disease resistances are often found to be controlled by a combination of race-specific resistance loci with large effects involved in pathogen recognition, and a large number of loci with small effects involved in basal resistance. Such a mixed QTL effect distribution can be found in maize *e.g.* for rust [13], Giberella ear rot [14,15] or to a lesser extent for Northern corn leaf blight [16,17].

For such traits, the assumption of normally distributed SNP effects underlying ridge regression, the most commonly applied WGP model, is severely violated. Heslot *et al.*[18] found for polygenic traits in several plant species only minor differences between ridge regression and models with different assumptions of the underlying distribution of SNP effects. However, these differences are expected to be much larger for traits controlled by only a few QTL. Recently, Clark *et al.*[19] simulated this situation under the assumption of the historical population structure of Holstein cattle. They found that under the assumption of either few common or few rare quantitative trait loci, a Bayesian variable selection model (BayesB) outperforms ridge regression by far. For Holstein cattle, Hayes *et al.*[20] found also the BayesA model to be superior to ridge regression in the case of coat color or milk-fat percentage.

Cattle differs greatly in its population structure and LD level from elite maize germplasm, which has faced severe genetic bottlenecks during domestication and the creation of genetically distinct heterotic pools to maximize exploitation of heterosis in hybrid breeding [21,22]. Hence, results from cattle might not be directly transferable to maize, for which little is known about WGP for traits with a simpler genetic architecture. Moreover, the genetic architecture of a trait is often unclear in crops. Especially if the trait has not yet been extensively dissected by linkage or association mapping, which might be the case for traits which gained only recently in importance such as nutritional properties, nutrient acquisition traits or traits related to climate change adaptation.

To fill this apparent gap of knowledge, we investigated WGP in a diverse collection of 289 maize inbred lines with traits which largely vary in their genetic architecture. To let the genetic basis differ as much as possible, we chose three highly polgygenic agronomic traits and three metabolites, each one controlled by a different major QTL explaining about 22% to 30% of genetic variance. With this empirical set-up, we asked the following questions: 

● To what extent do distinct WGP models differ in their prediction accuracies for a diversity panel of maize inbred lines if the genetic architecture of the trait changes dramatically?

● Are there guidelines for plants concerning the choice of the most promising WGP model?

## Methods

### Genetic material

The genetic material consisted of 289 maize inbred lines which were previously described in great detail [9,23-25]. The population constituted a global sample of elite breeding material from worldwide sources with a focus on North America and Europe and encompassed 285 lines from the Dent heterotic pool (Stiff-Stalk and non-Stiff-Stalk) and 4 from the European Flint pool, which served as check genotypes.

### Genotyping

The population was genotyped with the Illumina SNP chip MaizeSNP50 containing 56,110 SNPs [26]. Quality control preprocessing of SNPs was performed by eliminating SNPs that did not match the following criteria: (i) less than 10% missing values, (ii) minor allele frequency of greater than 2.5%, (iii) no more than three heterozygous genotypes, and (iv) unique allele assignment for the 22 replicated checks of genotype B73. A total of 38,019 SNPs remained and were used for further analysis. Linkage disequilibrium (LD) declined to *r*^2^=0.1 at approximately 500 kb with a mean LD between adjacent SNPs of 0.34 [9].

### Field trials

The population was phenotyped in six environments (three agroecologically diverse locations in the years 2008 and 2009) in Germany [25]. Briefly, the population was split into three maturity groups based on prior knowledge of their flowering time. In the trials of each of the three maturity groups, 100 genotypes, including five common check genotypes, were randomized in a 20 × 5 *α*-lattice design with two replications and were planted in 2-row plots. Plots were thinned to a final plant density of 100,000 plants/ha. The common check genotypes were used to adjust for potential differences in the soil fertility among trials in each environment.

### Metabolites

Leaf samples were collected in one location 33 d after sowing and processed using an established GC-MS method [27]. Genotypic means of Box-Cox transformed metabolite concentrations were obtained using a linear mixed model analysis including effects for field trial, replication, block, and batch. The whole metabolic profiling procedure including statistical analysis has been described in detail previously [9,23]. From the measured metabolite concentrations, we chose three highly heritable substances as model traits: dopamine, ribitol, and an unknown metabolite (719700-204). For each metabolite, we found in a genome-wide association (GWA) study a major metabolite QTL (mQTL) on different chromosomes after correcting for population structure and kinship [23]. For dopamine, the major mQTL was found on chromosome 9 and explained 28.9% of the genetic variance. For ribitol, the major mQTL was found on chromosome 10 and explained 22.1% oft the genetic variance. For the unknown metabolite, the major mQTL was found on chromosome 2 and explained 29.8% of the genetic variance. The metabolites were uncorrelated with each other (|*r*|≤0.10) and only weakly (|*r*|≤0.28) correlated with agronomic traits (Table 1).

**Table 1:** Phenotypic correlations among traits

**-**	**Plant height**	**Lignin content**	**Dopamine**	**Ribitol**	**719700-204**
Dry matter yield	0.62	0.32	-0.28	0.02	-0.24
Plant height	-	0.50	-0.17	0.07	-0.12
Lignin content	-	-	-0.20	-0.11	-0.08
Dopamine	-	-	-	-0.10	0.08
Ribitol	-	-	-	-	-0.03

### Agronomic traits

Dry matter yield of whole-plant biomass (t/ha) and plant height (m) were measured per field plot of the inbred lines. Lignin content was measured as acid detergent lignin (ADL) in the harvested plant material of the inbred lines using calibrated near-infrared spectroscopy (NIRS). The NIRS calibration model was built using phenotypic data from 20 inbred lines, 32 testcrosses and 3 hybrids grown in the same environments as the population of inbred lines analyzed in this study [24]. Heritability estimates and genotypic means were obtained using a one-step linear mixed model analysis as described previously [25]. Using a 1% Bonferroni corrected significance threshold, we could not find any significant SNP-trait association signal using the the same GWA model as for metabolites. Since population size, marker density, and heritabilites were sufficiently high for detecting QTL with large effects, the absence of any significant trait-SNP associations suggest a highly polygenic genetic architecture for the agronomic traits with no major QTL.

### Genome partitioning of the genetic variance

To further characterize the genetic architectures of the investigated traits irrespective of the significance thresholds for SNP-trait associations, we compared how the ten chromosomes contributed to the total genetic variance. Later on, we will use these results as a guideline for model selection based on trait architecture.

We adopted the approach of Yang *et al.*[28] to simultaneously estimate the genetic variance explained by each chromosome. In order to derive a guideline which is purely based on trait architecture and not on population structure artefacts, we additionally corrected for population structure by regressing the trait values on the first ten principal components. This linear model can be written as

(1) $$y=1\mu +Q\beta +\overset{10}{\underset{c=1}{\sum }}(Sg_{c})+e$$

where **y** is a vector with *n* trait values, **1**is a vector of 1’s, **Q**is a matrix of size *n*×10 containing the first 10 principal components calculated from SNP data with ***β*** containing the corresponding regression coefficients, **S** is an incidence matrix allocating components of **y**to components of **g**_*c*_, which is a vector of length *n* with random genotypic effects attributable to chromosome *c* with $\left(g_{c}∼N(0,G_{c}\sigma_{\mathrm{gc}}^{2})\right)$ and $\left(G_{c}=Z_{c}Z_{c}^{T}/p_{c}\right)$ where **Z**_*c*_ is a matrix of size *n*×*p*_*c*_with standardized levels of SNP alleles on chromosome *c*. Vector **e** contains normally distributed residuals with $\left(e∼N(0,I\sigma_{e}^{2})\right)$. The genetic variance contributed by chromosome *c* was then estimated as $\left(\sigma_{\mathrm{gc}}^{2}/(\overset{10}{\underset{c=1}{\sum }}(\sigma_{\mathrm{gc}}^{2})+\sigma_{e}^{2})\right)$.

Variance components were estimated by restricted maximum likelihood (REML) using ASReml-R 3 [29]. Since matrices **G**_*c*_were often found to be singular, we used the algorithm of Higham [30] implemented in the function nearPD of the R-package Matrix [31], to approximate the nearest positive definite matrices.

### WGP models

We investigated five WGP models that have been recently advocated in the literature for this purpose [4,32-34].

All based upon the classical regression set-up

(2) y=1μ+Zu+e

where **y** is a vector with *n* trait values, *μ* is the overall mean, **1** is a vector with 1’s, **Z** is the *n*×*p* matrix of standardized values of SNP alleles, **u** is a vector with SNP effects, and **e** is a vector of residuals with $\left(e∼N(0,I\sigma_{e}^{2})\right)$. Depending on the trait, a combination of genotypic and phenotypic information was available for 276 to 280 genotypes which were used for WGP.

#### RR-BLUP

**Figure 1:**
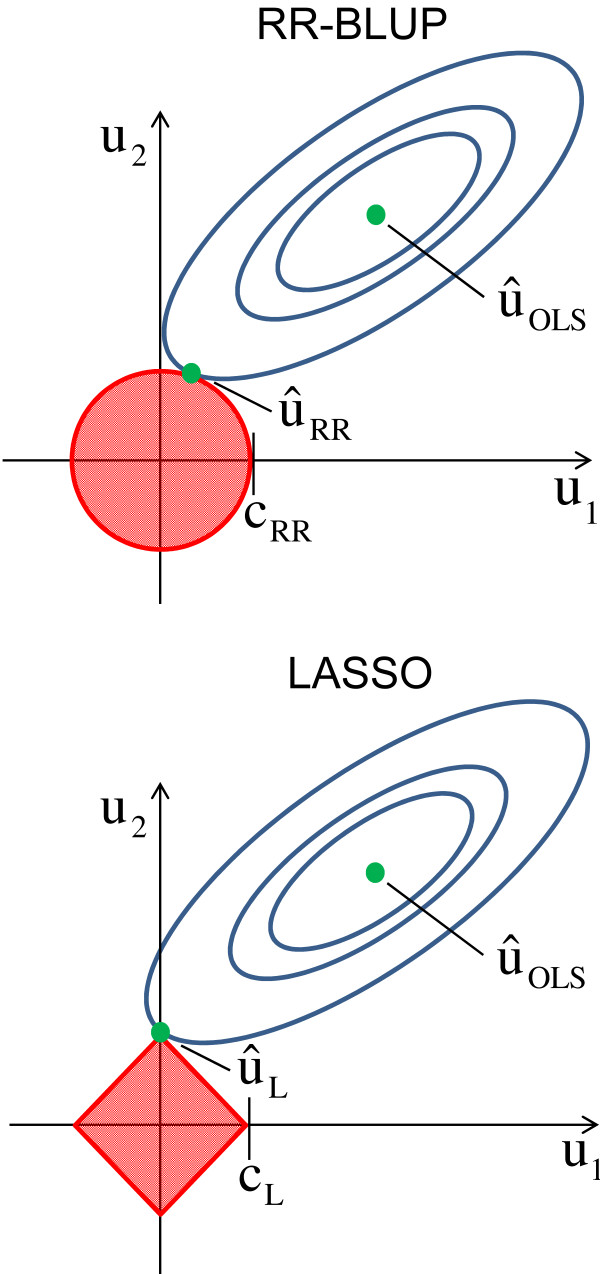
**Visualization of the RR-BLUP estimator (**$\left({\hat{u}}_{\text{RR}}\right)$**) and the LASSO estimator (**$\left({\hat{u}}_{\text{L}}\right)$**) as solutions to a least-squares problem with different penalization [**[38]**,**[39]**].** We illustrate a two-dimensional case. The blue ellipses show the contours of the RSS function around the ordinary least-square solution ($\left({\hat{u}}_{\text{OLS}}\right)$). The ridge estimator is the point at which the innermost elliptical contour touches the circular ridge penalty $\sqrt{u_{1}^{2}+u_{2}^{2}}<c_{\text{RR}}$. The LASSO estimator is the point at which the innermost elliptical contour touches the diamond shaped LASSO penalty |*u*_1_| + |*u*_2_|<*c*_L_. Contrary to the ridge penalty, the LASSO penalty allows estimations to be exactly zero.

Ridge regression (RR) tackles the *p*≫*n*problem in WGP by minimizing the residual sum of squares (RSS=(**y**−**1***μ*−**Zu**)^*T*^(**y**−**1***μ*−**Zu**)) by bounding the Euclidean (*L*_2_) norm of **u** to a constraint: $||u|{|}_{2}=\sqrt{\overset{p}{\underset{i=1}{\sum }}u_{i}^{2}}<c_{\mathrm{RR}}$ which leads to a homogeneous shrinkage of all SNP effects towards zero (Figure 1). The RR estimator is given by

(3) $${\hat{u}}_{\mathrm{RR}}=\arg\underset{u}{\min}(\mathrm{RSS}+\lambda_{\mathrm{RR}}||u|{|}_{2}).$$

The Lagrangian multiplier *λ*_RR_is a regularization parameter which controls the amount of shrinkage. It can be estimated as $\left(\lambda_{\mathrm{RR}}=\sigma_{e}^{2}/\sigma_{u}^{2}\right)$ by regarding **u** as random effects with $\left(u∼N(0,I\sigma_{u}^{2})\right)$ with $\sigma_{u}^{2}$ being the SNP effect variance estimated by REML. In this setting, $\left({\hat{u}}_{\mathrm{RR}}\right)$ is equivalent to the best linear unbiased predictor (BLUP) of **u**[35,36].

For computational convenience, RR-BLUP can be transformed to a mathematically equivalent model

(4) y=1μ+Sg+e

with **g** being a vector of random genotype effects with var(**g**) = $\left(G\sigma_{g}^{2}\right)$ and whole-genome relationship matrix **G**=**ZZ**^*T*^/*p*. The solution vector of SNP effects can then be obtained as $\left({\hat{u}}_{\mathrm{RR}}=Z^{T}G^{-1}\hat{g}\right)$[37]. Here, **G** is an inner-product kernel which allows to perform all computations in the space of *n* genotypes instead of *p* SNPs, a shortcut which is well established in the field of kernel-based machine learning [38].

#### LASSO

As an alternative to ridge regression, it was suggested to use an *L*_1_ penalty to tackle the *p*≫*n*problem [39]. This estimator was termed least absolute shrinkage and selection operator (LASSO) and has recently been suggested for whole-genome prediction [32,40,41]. The estimator is given by

(5) $${\hat{u}}_{L}=\arg\underset{u}{\min}(\mathrm{RSS}+\lambda_{L}||u|{|}_{1})$$

which bounds the Manhattan (*L*_1_) norm of **u** to a constraint: $\left(||u|{|}_{1}=\overset{p}{\underset{i=1}{\sum }}|u_{i}|<c_{L}\right)$. The LASSO penalty is a diamond shaped constraint which allows not only to shrink coefficients towards zero but to set some coefficients to exactly zero (Figure 1). Unlike RR, LASSO, cannot be ’kernelized’, *i.e.*, it is not possible to transform the LASSO estimator into an equivalent kernel regression problem in the space of *n* genotypes [38]. Hence, LASSO regression has to be carried out with the full set of SNPs. Here, we used the R package glmnet, a fast implementation using cyclic coordinate descent to compute the complete LASSO path solution [42].

#### Elastic net

The LASSO penalty is known to be somewhat indifferent to the choice among a set of strong but correlated variables. The RR penalty, on the other side, tends to shrink the coefficients of correlated variables toward each other [38]. The elastic net (EN) estimator is a compromise which can be written as

(6) $${\hat{u}}_{\mathrm{EN}}=\arg\underset{u}{\min}(\mathrm{RSS}+\lambda_{\mathrm{EN}}(\alpha ||u|{|}_{1}+(1-\alpha )||u|{|}_{2})),$$

and is a weighted mixture between the RR penalty (*α*=0) and the LASSO penalty (*α*=1) [33]. While the RR penalty encourages highly correlated variables to be averaged, the LASSO penalty encourages a sparse solution [38]. We again used the implementation in glmnet and performed a grid search to find the combination of *α* and *λ*_EN_, which yielded the lowest mean squared error in the training population.

#### Reproducing kernel Hilbert space (RKHS) regression

The theory of RKHS regression is rooted in the field of kernel-based machine-learning [38] and has recently been advocated for whole-genome prediction [34]. The approach uses equation 4 but replaces the inner-product matrix **G** with a kernel matrix **K**. The motivation behind RKHS regression lies in the ability to effectively perform non-linear regression in a higher-dimensional feature space so it might capture non-additive genetic effects, if present. Here, we used a Gaussian kernel on genetic distances with $K_{\mathrm{ij}}=\exp(-{\mathrm{GD}}_{\mathrm{ij}}/\theta^{2})$, where GD_*ij*_ is the modified Rogers’ genetic distance (Euclidean distance scaled to fall between 0 and 1) between genotype *i* and *j*, and *θ* is a smoothing parameter which controls the rate of decay of *K*_*ij*_ with increasing genetic distance. The optimum value for *θ*was chosen from a sequence from 0.1 to 100 at which the maximum likelihood was obtained.

#### BayesB

As a Bayesian approach, we used a modified version of BayesB, which has a prior assumption that the SNP effects are *t*-distributed with a point-mass at zero [4]. Following the suggestions of Yang and Tempelman [43], we modeled several hyperparameters as uncertain too. Details of the priors used can be found in Table 2. To fit the model, we ran the Gibbs-sampler for 50,000 iterations. The first 5,000 iterations were discarded as burn-in and only samples from every 10^th^ post burn-in iteration were stored. For computational convenience, we reduced the number of markers to 5,000 SNPs for which we did not observe any decline in prediction accuracy up to the numerical precision reported in this study.

**Table 2:** Priors used for BayesB

**Parameter**	**Prior**
*u*_*i*_	$N(0,\sigma_{\mathrm{ui}}^{2})$
$\left(\sigma_{\mathrm{ui}}^{2}|v_{u},S_{u}^{2}\right)$	$\left\{\begin{array}{l} 0\;\;\;\;\;\;\text{with probability}\;\Pi_{u},\\ \chi^{-2}(v_{u},S_{u}^{2})\;\;\text{with probability}\;(1-\Pi_{u}) \end{array}\right)$
*v*_*u*_	Gamma(*k*=5,*θ*=2)
$\left(S_{u}^{2}\right)$	Gamma(*k*=0.1,*θ*=10)
*Π*_*u*_	Beta(*α*=7,*β*=3)
$\left(\sigma_{e}^{2}\right)$	$\left(\chi^{-2}(v_{e},S_{e}^{2}=\hat{\sigma_{e}^{2}}(v_{e}-2)/v_{e})\right)$
	*v*_*e*_=4.001, $\left(\hat{\sigma_{e}^{2}}\right)$ estimated with REML

### Validation

A five-fold cross-validation scheme was applied and repeated 20 times. In each repetition, the dataset was divided into 5 disjoint subsets of genotypes whereas one subset served as the validation set and the other four subsets served as the training population to estimate the model parameters for predicting the left-out genotypes in the validation set. In each of the five rounds, the Pearson correlation between the observed and predicted phenotypic values was calculated. The procedure was repeated twenty times to yield 100 cross-validation runs. The predictive ability was then calculated as the Pearson correlation ($\left(r_{\left(y,\hat{y}\right)}\right)$) between the observed (**y**) and predicted ($\hat{y}$) phenotypic values. The ’prediction accuracy’ estimates the correlation ($r_{\left(g,\hat{g}\right)}$) between the predicted ($\hat{g}$) and unobserved true genetic values (**g**) and was calculated by $r_{\left(g,\hat{g}\right)}=r_{\left(y,\hat{y}\right)}/h$ where *h* is the square root of the heritability on a line-mean basis for the agronomic traits. For metabolites, the square root of the estimated repeatability was used.

## Results

**Figure 2:**
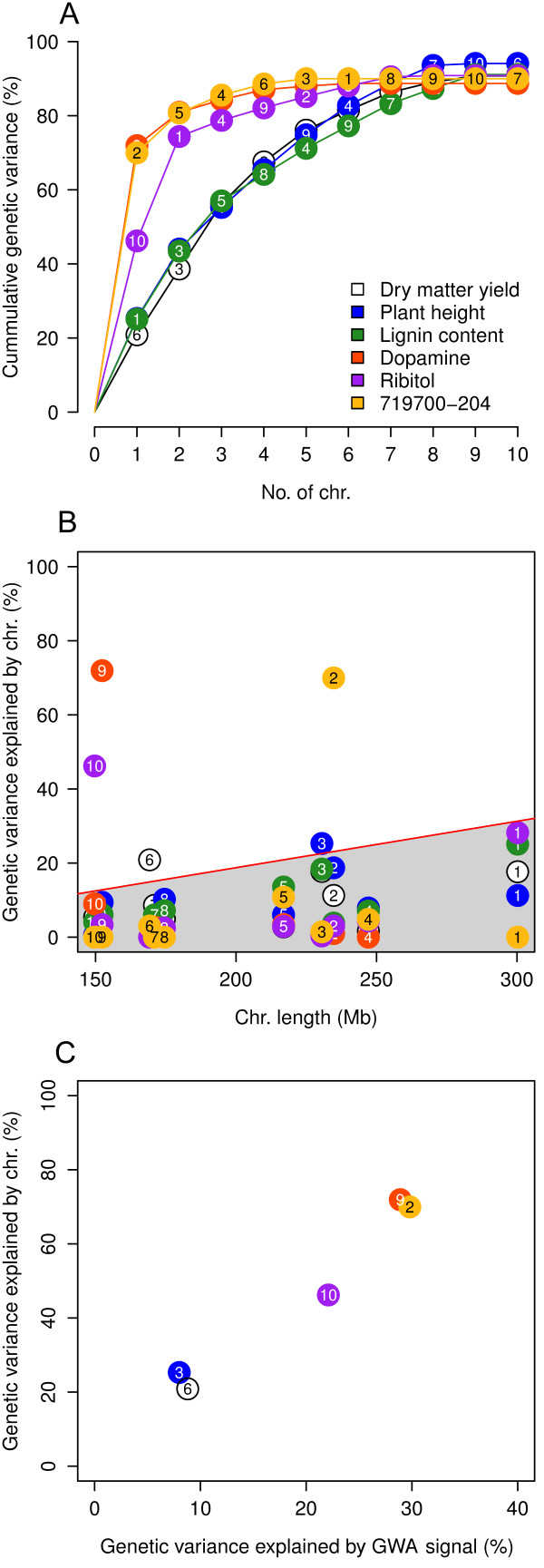
**Characterization of the genetic architecture of different traits by genome partitioning of the genetic variance. **(**A**) Cummulative genetic variance explained by individual chromosomes. (**B**) Genetic variance explained by each chromosome (number in points). The chromosomes containing either major mQTL for metabolites or putative minor QTL for agronomic traits lie above the red line. (**C**) Genetic variance explained by chromosomes plotted against the genetic variance explained by the GWA signals on these chromosomes.

The contribution of the individual chromosomes to the genetic variance differed largely between metabolites and agronomic traits (Figure 2). For the metabolites, the chromosomes containing the major mQTL (chromosome 9 for dopamine, chromosome 10 for ribitol, and chromosome 2 for the unknown metabolite) captured by far the largest portion of the genetic variance, leaving the remaining genetic variance equally distributed over the remaining chromosomes.

For the agronomic traits, the total genetic variance was largely uniformly distributed over all chromosomes. Using the same GWA model as for the metabolites [23], we found that for dry matter yield and plant height, the chromosomes which captured the largest portion of genetic variance contain the strongest GWA signals. However, in no instance was the 1% Bonferroni corrected significance threshold surpassed (dry matter yield: chr. 6, *P*=4.06×10^−6^, position 139,284,469, explained genetic variance 8.8%; plant height: chr. 3, *P*=8.6×10^−6^, position 163,617,228, explained genetic variance 8.0%).

When excluding chromosomes containing either these two association signals or major mQTL, we observed a tendency that longer chromosomes captured more genetic variance than shorter ones (Figure 2B). This trend was significant (*P*<0.10) for lignin content (*r*=0.88,*P*=7.0×10^−4^) and dry matter yield (*r*=0.60,*P*=0.09). The grey area in Figure 2B was therefore regarded as the range in chromosomal genetic variance explainable by the length of the chromosome. On the other side, the genetic variance contributed by the left-out chromosomes was highly correlated (*r*=0.98,*P*=0.003) with the explained genetic variance of the individual SNPs found by GWA mapping (Figure 2C).

The total genetic variance summed over all chromosomes amounted to 0.91 for dry matter yield, 0.94 for plant height, 0.91 for lignin content, 0.89 for dopamine, 0.91 for ribitol, and 0.90 for the unknown metabolite. These values were close to the heritabilities and repeatabilities obtained from the phenotypic analysis (Table 3).

**Table 3:** **Prediction accuracies**$\left(r_{\left(g,\hat{g}\right)}\right)$**and their standard deviations (s.d.) for different WGP models**

**Trait**	***h***^***2***^	**RR-BLUP**		**LASSO**		**Elastic net**		**RKHS**		**BayesB**	
		$r_{\left(g,\hat{g}\right)}$	**s.d.**	$r_{\left(g,\hat{g}\right)}$	**s.d.**	$r_{\left(g,\hat{g}\right)}$	**s.d.**	$r_{\left(g,\hat{g}\right)}$	**s.d.**	$r_{\left(g,\hat{g}\right)}$	**s.d.**
Dry matter yield	0.93	0.61	0.07	0.51	0.11	0.56	0.08	0.61	0.07	0.59	0.08
Plant height	0.97	0.57	0.09	0.45	0.11	0.48	0.11	0.57	0.09	0.56	0.08
Lignin content	0.88	0.69	0.07	0.60	0.08	0.60	0.10	0.68	0.07	0.58	0.09
Dopamine	0.97	0.74	0.06	0.79	0.06	0.79	0.06	0.74	0.07	0.75	0.06
Ribitol	0.95	0.49	0.12	0.61	0.10	0.63	0.10	0.50	0.10	0.50	0.11
719700-204	0.96	0.79	0.06	0.82	0.05	0.82	0.05	0.80	0.05	0.80	0.08

Results are averaged over all 100 cross-validation runs. For the agronomic traits, *h*^2^is the heritability on a line-mean basis and for the metabolites, the repeatability is shown.

Prediction accuracies of WGP ranged between 0.45 and 0.82 with standard deviations between 0.05 to 0.12 across traits and models (Table 3). The largest differences in accuracies between models ranged from 0.05 to 0.14 for the same trait. Between RR-BLUP and RKHS, we found no difference in the prediction accuracies above 0.01 for any trait.

For agronomic traits, prediction accuracies were highest for RR-BLUP with a drop of 0.09 to 0.12 if LASSO or elastic net was used and with a drop of 0.01 to 0.11 if BayesB was used.

**Figure 3:**
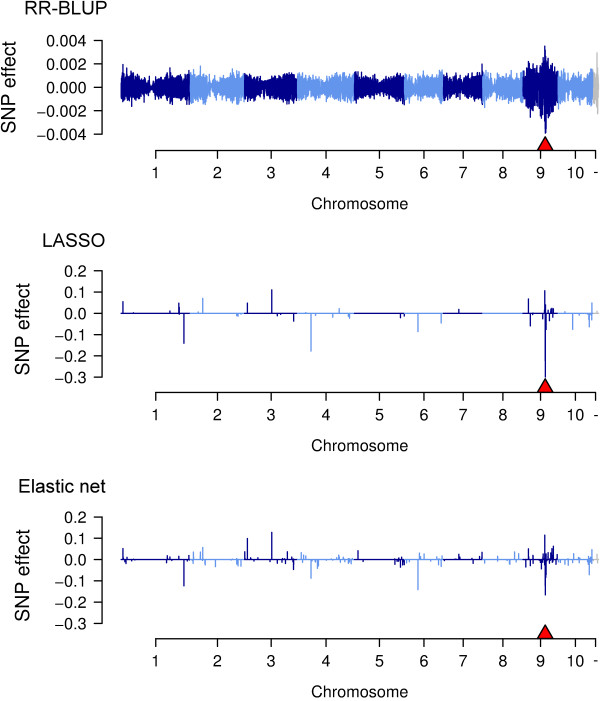
**SNP effects for dopamine obtained by using either RR-BLUP, LASSO, or the elastic net model.** The position of the mQTL is indicated as a red triangle.

The ranking of the prediction accuracies for the WGP models was reverse for metabolites. Here, prediction accuracies were highest for LASSO or elastic net with a drop of 0.05 to 0.14 when using RR-BLUP. For metabolites, no differences in the prediction accuracies above 0.01 were observed between RR-BLUP and BayesB. For dopamine and the unknown metabolites, the mQTL were precisely found with LASSO, elastic net and also RR-BLUP (Figure 3). For all three models, their largest absolute SNP effect matched exactly with the SNP identified by GWA mapping. However, the three models differed drastically in their sparsity in SNP effects, and the distance over which the mQTL effect was distributed. Whereas the mQTL effects declined sharply with LASSO or elastic net, they were diluted over a much longer distance with RR-BLUP.

## Discussion

We found in a diverse panel of elite maize inbred lines that prediction accuracies obtained with five different WGP models were remarkable similar, even for traits with drastically deviating genetic architecture. Our results suggest that small gains in accuracies (up to 0.14) can be gained if the WGP model is selected according to the genetic architecture underlying the trait.

Recently, Heslot *et al.*[18] reported similar small differences for seven parametric WGP models when comparing them for different presumable highly polygenic agronomic traits over eight datasets of barley, *Arabidopsis thaliana*, maize, and wheat. For the metabolites, however, our results differ from those obtained from Clark *et al.*[19], who investigated the influence of genetic architecture on prediction accuracies achieved by RR-BLUP or BayesB. Whereas these authors found only slight differences for simulated traits with a genetic architecture close to the infinitesimal genetic model, BayesB outperformed RR-BLUP by an increase in prediction accuracy of ≈0.4 if the trait is controlled by either a few common or a few rare QTL. Simulation also predicted a drop in prediction accuracy in case of RR-BLUP for traits controlled by a small number of QTL [44]. Although LASSO, elastic net, and BayesB showed higher accuracies compared to RR-BLUP for metabolites, we found the differences to be remarkable small in case of LASSO or elastic net and negliable in the case of BayesB.

One major reason of the minor differences in prediction accuracies among the different models lies in the high level of LD found in elite breeding germplasm of maize. Our results suggest that with this level of LD (*r*^2^=0.1 at ≈ 500 kb), accuracies are quite similar irrespective whether the effect of large QTL are precisely captured (as in the case of LASSO, elastic net, or BayesB) or spread over a larger region (as in the case of RR-BLUP and RKHS). Since our population was highly diverse for elite maize germplasm in Europe, it is unlikely that breeders are confronted with lower levels of LD unless they work with highly exotic germplasm for which LD has been reported to decline within 5-10 kb [45].

Moreover, the high similarity of RKHS and RR-BLUP suggest that either (i) non-additive, epistatic genetic effects are not present, (ii) these are so small that they are negligible in WGP for the investigated traits, or (iii) RKHS regression is unable to capture them. In either case, for prediction purposes RKHS does not seem to yield any advancements over RR-BLUP for situations comparable to our germplasm and traits. Dominance, as another source of non-additive genetic, effects cannot be present in the inbred lines investigated in this study. For predicting heterozygeous F_1_ maize hybrids, however, it has been shown that modeling dominance effects can result in higher prediction accuracies [8].

Although BayesB reached for 5 of the 6 traits a higher prediction accuracy than the worst model, we cannot recommend it because of the excessively larger computation time and the negliable differences in prediction accuracies compared with RR-BLUP in case of the metabolites as the result of probably only sampling error.

We found the approach to partition genetic variance over chromosomes useful for guiding the breeder which WGP model to prefer in the case of little or no prior knowledge on the genetic architecture. Whereas for the agronomic traits an approximately linear increase of cumulative explained genetic variance matched with a superiority of the *L*_2_ penalty (RR-BLUP), the *L*_1_penalty (LASSO) or a mixture of both penalties (elastic net) performed better in the case of the metabolites with a strong convex curve curvature (Figure 2A). Although for dry matter yield and plant height, barely significant association signals with a proportion of explained genetic variance <9*%* led to a chromosomal genetic variance slightly above the range expected from length of the chromosome (Figure 2B), these effects were too small to justify the use of the elastic net or LASSO.

As an alternative to this approach, Hayes *et al.*[20] estimated successively the genetic variance explained by each chromosome segment and compared it with the genetic variance captured by the remaining part of the genome. To correct for the non-independence of neighbouring segments, they applied a bias correction using an expectation maximization (EM) algorithm. Such a correction is not necessary if the variance components for all chromosomes are estimated simultaneously as applied in this study; this is a further advantage besides its straightforward implementation using standard mixed model software packages such as ASReml.

## Conclusions

Our empirical data of WGP in a large panel of diverse maize inbred lines suggest that (i) different WGP models differ only slightly in their prediction accuracies, irrespective of the number and effects of QTL found in association analysis, (ii) small gains in prediction accuracies can be obtained if the WGP model is selected according to the genetic architecture of the trait, (iii) genome partitioning of genetic variance offers a straightforward approach for model selection if the genetic architecture is unknown. The question of which WGP model to choose is therefore not expected to hamper implementation of WGP in maize breeding.

## Competing interests

The authors declare that they have no competing interests.

## Authors’ contributions

CR analyzed the data and wrote the manuscript. AEM supervised the research. FT wrote the C code for implementing BayesB. All authors read and approved the final manuscript.
